# Dopamine: A Modulator of Circadian Rhythms in the Central Nervous System

**DOI:** 10.3389/fncel.2017.00091

**Published:** 2017-04-03

**Authors:** Kirill S. Korshunov, Laura J. Blakemore, Paul Q. Trombley

**Affiliations:** ^1^Program in Neuroscience, Florida State University,Tallahassee, FL, USA; ^2^Department of Biological Science, Florida State University,Tallahassee, FL, USA

**Keywords:** circadian rhythms, dopamine, retina, olfactory bulb, striatum, midbrain, hypothalamus, Parkinson’s disease

## Abstract

Circadian rhythms are daily rhythms that regulate many biological processes – from gene transcription to behavior – and a disruption of these rhythms can lead to a myriad of health risks. Circadian rhythms are entrained by light, and their 24-h oscillation is maintained by a core molecular feedback loop composed of canonical circadian (“clock”) genes and proteins. Different modulators help to maintain the proper rhythmicity of these genes and proteins, and one emerging modulator is dopamine. Dopamine has been shown to have circadian-like activities in the retina, olfactory bulb, striatum, midbrain, and hypothalamus, where it regulates, and is regulated by, clock genes in some of these areas. Thus, it is likely that dopamine is essential to mechanisms that maintain proper rhythmicity of these five brain areas. This review discusses studies that showcase different dopaminergic mechanisms that may be involved with the regulation of these brain areas’ circadian rhythms. Mechanisms include how dopamine and dopamine receptor activity directly and indirectly influence clock genes and proteins, how dopamine’s interactions with gap junctions influence daily neuronal excitability, and how dopamine’s release and effects are gated by low- and high-pass filters. Because the dopamine neurons described in this review also release the inhibitory neurotransmitter GABA which influences clock protein expression in the retina, we discuss articles that explore how GABA may contribute to the actions of dopamine neurons on circadian rhythms. Finally, to understand how the loss of function of dopamine neurons could influence circadian rhythms, we review studies linking the neurodegenerative disease Parkinson’s Disease to disruptions of circadian rhythms in these five brain areas. The purpose of this review is to summarize growing evidence that dopamine is involved in regulating circadian rhythms, either directly or indirectly, in the brain areas discussed here. An appreciation of the growing evidence of dopamine’s influence on circadian rhythms may lead to new treatments including pharmacological agents directed at alleviating the various symptoms of circadian rhythm disruption.

## Introduction

Our daily lives operate on 24-h cycles that are regulated by circadian rhythms. Circadian rhythms synchronize our biological processes, including body temperature, hunger, sleep, gene transcription, and sensory perceptions, to manifest and repeat during specific times each day. The field of circadian rhythm research continues to expand, and it is becoming apparent just how crucial it is to our health to follow these daily rhythms. For example, those in today’s constantly working society often experience disruptions in their natural circadian rhythms from nightshifts, jet lag, social jet lag (often experienced by adolescents and high-school students who wake up at an earlier time than their biological clocks demand), and overall short or fragmented nighttime sleep. These disruptions may increase risks of developing cardiovascular disease (Dominguez-Rodriguez et al., 2009), cancer (Schernhammer et al., 2006), obesity (McHill et al., 2014), and metabolic syndrome (Sookoian et al., 2007; Dochi et al., 2009). Therefore, it is becoming crucial to understand not only how circadian rhythms regulate daily biological processes, but also their molecular framework. Such an understanding may be beneficial to formulating treatment strategies for the above conditions, as well as redefining societal expectations and pressures to work during the biological night.

Human and vertebrate circadian rhythms are synchronized by the master circadian pacemaker, the SCN, which resides in the hypothalamus above the optic chiasm. The SCN entrains circadian rhythms throughout the body, and it does so by receiving light information from the melanopsin-expressing ipRGCs, via the retinohypothalamic tract (Gooley et al., 2001; Hattar et al., 2006; see Figure 1 in Mendoza and Challet, 2014). On a molecular level, the SCN and other cells maintain their own circadian rhythms through feedback loops between canonical circadian clock genes and proteins (here on referred to as “clock” genes and proteins). Clock genes include *Bmal1*, *Clock*, *Per1* and *2*, *Cry1* and *2*, *Npas2*, and *Rev-erb*α, and their protein derivatives BMAL1, CLOCK, PER 1 and 2, CRY 1 and 2, NPAS2, and REV-ERBα (Lowrey and Takahashi, 2011; Mohawk et al., 2012). The core circadian feedback loop formed from interactions between these genes and proteins is as follows: CLOCK and BMAL1 bind to the promoter regions of *Per* and *Cry*, initiating transcription of these genes; the protein products, PER and CRY, form a complex that enters the cell’s nucleus and represses the transcriptional activity of CLOCK and BMAL1, subsequently stopping *Per* and *Cry* transcription (Lowrey and Takahashi, 2011; Mohawk et al., 2012). This loop repeats every 24 h. Each step of the loop is synchronized (entrained) to a specific time point. Light and other environmental cues entrain each circadian mechanism to a specific ZT (“time giver”). Without light or other external cues, the internal clock oscillates on what is called CT. CLOCK and BMAL1 also target the promoter regions of other genes, which drive other circadian feedback loops and biological processes, depending on the cell. Along with the SCN, other neuromodulators contribute to the proper cycling of clock genes and proteins, which overall keeps these biological processes running on time.

Dopamine, a neurotransmitter well known for regulating movement, reward, and learning, is emerging as one of the neuromodulators of central and peripheral circadian rhythms. Studies of both the SCN and peripheral brain areas have shown explicit and potential evidence of DA modulating, or being modulated by, neuronal clock genes, proteins, and rhythms. This review focuses on five of those brain areas: the retina, OB, striatum, midbrain, and hypothalamus (See Supplementary Table 1).

Dopamine is a well-known modulator of circadian rhythms in the retina. In particular, the circadian release of vertebrate retinal DA (either endogenously expressed in the interplexiform, amacrine, or both cells, depending on the species) allows for proper light adaptation and transmission of light information to the SCN, via the melanopsin-expressing ipRGCs (Gooley et al., 2001; Hattar et al., 2006; Popova, 2014; Prigge et al., 2016). The neuronal circuitry of the retina is morphologically similar to that of the OB, and the OB also expresses DA in the glomerular interneurons. Our lab has found a diurnal variation in DA release in the OB (Corthell et al., 2013), which may suggest that DA is involved in neuromodulation of OB circadian rhythms (see Mendoza and Challet, 2014). In the dorsal striatum, dopaminergic input is required for proper modulation of PER2 (Hood et al., 2010), and DA receptors regulate clock gene expression in the striatum *in vitro* (Imbesi et al., 2009). Additionally, TH (the rate-limiting enzyme in DA synthesis) has diurnal variation in the striatum and dopaminergic projecting neurons from the midbrain (Webb et al., 2009), and TH is also regulated by clock genes in the midbrain (McClung et al., 2005; Webb et al., 2009; Chung et al., 2014; Sidor et al., 2015). Lastly, we include the hypothalamus because it houses the SCN and because of its circadian-controlled homeostatic functions, including the circadian-like modulation of PRL release by the dopaminergic tuberoinfundibular neurons (Freeman et al., 2000; Bertram et al., 2010). This review does not explore DA modulation of circadian rhythms in the hippocampus. However, the hippocampus’ circadian clock may be involved in mood regulation and neurogenesis (see review by McCarthy and Welsh, 2012), which can be potential targets of DA’s mesolimbic pathway.

To validate the importance of DA to circadian rhythms in humans and rodents, the neurodegenerative disorder, PD, is also explored. Affecting millions worldwide, PD destroys dopaminergic neurons of the SNc, leading to common motor symptoms (e.g., bradykinesia, rigidity, tremor) (Carlsson, 1972). Among classes of DA receptors, D_1_ and D_2_ receptors play a central role in the pathogenesis of PD (Videnovic and Golombek, 2013). Dysfunction of dopaminergic populations in brain regions including the midbrain, striatum, retina, OB, and hypothalamus also may lead to other circadian symptoms of PD such as altered locomotor activity (Fifel and Cooper, 2014), sleep disturbances (Turjanski et al., 1999; Eisensehr et al., 2003; Lima et al., 2007; Chaudhuri and Schapira, 2009; Lima, 2013; Videnovic and Golombek, 2013), visual dysfunction (see Archibald et al., 2009; Chaudhuri and Schapira, 2009; Popova, 2014), olfactory dysfunction/hyposmia (Doty et al., 1988; Huisman et al., 2004; Ross et al., 2008; Lelan et al., 2011; Doty, 2012), and disruptions to cyclic PRL release (Murri et al., 1980; Bellomo et al., 1991; Winkler et al., 2002). Recent evidence suggests that PD may affect circadian rhythms and their cellular mechanisms differently in the peripheral hypothalamus than in the SCN (Gravotta et al., 2011; Kudo et al., 2011; Hayashi et al., 2013; Mattam and Jagota, 2015).

Dysregulation and alterations in circadian genes are commonly observed in people and animal models with PD (Cai et al., 2010; Kudo et al., 2011; Hayashi et al., 2013; Anderson and Maes, 2014). Oxidative stress, a potential inducer of PD, may be regulated by clock genes (see Anderson and Maes, 2014). *Per1* and *Per2* regulate cAMP activity, which regulates the AHr (Anderson and Maes, 2014). A dysregulation of cAMP, potentially through the desynchronization of *Per1* and *Per2*, can lead to an AHr-induced reduction in melatonin synthesis (Anderson and Maes, 2014). A decrease in melatonin, which is an antioxidant, can lead to mitochondrial dysfunction and atrophy of SNc’s dopaminergic neurons (Anderson and Maes, 2014), potentially leading to PD-like symptoms.

In regard to studies of PD, it should be noted that limitations to various animal models of this disease exist (see Fifel and Cooper, 2014). For example, most transgenic mouse models of PD do not show a selective and/or progressive neurodegeneration of DA neurons (Dawson et al., 2010). In addition, the acute nature of the insult on DA neurons in neurotoxin-based models differs from the slow, progressive, age-dependent neurodegeneration of DA neurons seen in PD (Maetzler et al., 2009; Chesselet and Richter, 2011). Thus, many of these models lack the key motor and non-motor disruptions observed in PD patients (see Fifel and Cooper, 2014). That being said, data from studies using animal models of PD are included in this review to fill gaps in data from human studies.

The study of DA as a neuromodulator can provide additional understanding to what is already known about the molecular and neuronal mechanisms that drive the circadian rhythms of the brain and other areas. Furthermore, this knowledge could lead to pharmacological therapies aimed at correcting abnormal neuronal mechanisms in those with disrupted circadian rhythms, and, potentially, pharmacological therapies to alleviate symptoms of PD.

## Retina

The retina (see Figure 1 in Popova, 2014) is the first region of the nervous system to receive and process light, before sending the visual information to the rest of the brain. The retina supplies light information to the SCN via the retinohypothalamic pathway, and it is critical for entrainment of the body’s circadian rhythms (Gooley et al., 2001; Hattar et al., 2006). The neurons of the retina, as those of the OB, can also maintain the synchrony of their circadian rhythms independent of the SCN (Doyle et al., 2002a,b; Granados-Fuentes et al., 2006; Ruan et al., 2008; Jaeger et al., 2015), and DA has been directly linked to the regulation of circadian rhythms of retinas *in vitro* (Doyle et al., 2002a,b; Ruan et al., 2008).

### Daily Rhythm of Retinal Dopamine Content Depends on Retinal Melatonin

The mammalian and fish retinas have a daily rhythm of DA content, which peaks during the daytime hours and falls during the nighttime hours (Doyle et al., 2002a,b; Ribelayga and Mangel, 2003). This retinal DA rhythm appears to be dependent on melatonin, a hormone that helps to induce sleep during darkness (Doyle et al., 2002a). In the Doyle et al. (2002a) study, a mouse strain (C57BL/6J) was used that was unable to produce endogenous melatonin, and, as a result, the circadian rhythm of retinal DA levels was lost in constant darkness. With the application of exogenous melatonin to the mouse retina, DA rhythmicity in constant darkness was restored (Doyle et al., 2002a). Thus, there may be a mutual inhibition between DA and melatonin, which leads to daily rhythmicity in the retina (Iuvone, 1986; Cahill and Besharse, 1991, 1995; Doyle et al., 2002a).

On the other hand, the rhythm of DA content in the retina may not depend on the integrity of the retina’s photoreceptor cells (Doyle et al., 2002b). The rat strain RCS/N-*rdy*, whose photoreceptors degenerate after 18 days of age, had an unaltered daytime rhythm of DA, both in 12-h light/dark cycles and in constant darkness (Doyle et al., 2002b). In the absence of photoreceptors, the unaltered rhythmicity of retinal DA may be entrained by ipRGCs, as these cells send glutamatergic signals to postsynaptic dopaminergic cells (Prigge et al., 2016). This Doyle et al. (2002b) study also confirmed that retinal contents of DA and its metabolites are higher during the daytime rather than nighttime. This finding is consistent with that of Di Paolo et al. (1987), who showed that postmortem human retinas had higher DA concentrations when the person died during the daytime rather than the nighttime (Di Paolo et al., 1987). This is also consistent with the finding that light increases retinal TH activity and DA turnover in the rat retina (Iuvone et al., 1978).

### Dopamine Receptors’ Vast Effects on Retinal Light Adaptation and Circadian Rhythms

The vertebrate retina expresses two classes of DA receptors: D_1_-like receptors (which includes D_1_ and D_5_ receptors) and D_2_-like receptors (which includes D_2_, D_3_, and D_4_ receptors, although D_3_ may not be present in the retina) (Witkovsky, 2004; Popova, 2014). D_1_ receptors are found on horizontal, amacrine, and bipolar cells, while D_5_ receptors are found in the retinal pigment layer (Witkovsky, 2004). Some of the D_2_ receptors function as autoreceptors for DA neurons, while D_4_ receptors are found on rods and cones photoreceptors, at least in mice (Witkovsky, 2004).

Evidence suggests that the D_1_ receptor, but not the D_2_ receptor, influences the PER2 protein of the retina. Exposing mouse retinal explant cultures to 1-h pulses of light during CT 13 and CT 19.5 (retinal subjective night) delays retinal PER2 expression by 2.3 h at CT13 and by 1.5 h at CT 19.5 (Ruan et al., 2008). The *in vitro* expression of retinal PER2 is also affected by the D_1_ receptor antagonist, SCH-23390, and a 1-h pulse of light at CT 13 (Ruan et al., 2008). SCH-23390 applied during the 1-h light pulse decreased the PER2 expression delay significantly compared to the effects of light exposure alone (Ruan et al., 2008). Without light exposure, application of the D_1_ receptor agonist, SKF38393, at CT 13 induced a delay of PER2 expression by 1 h (Ruan et al., 2008). Conversely, activating the D_2_ receptor with the agonist quinpirole, applied at CT 13, does not affect the timing of the PER2 expression (Ruan et al., 2008).

Activation of the D_2_ receptor induces *Per1* transcription through the recruitment of the CLOCK:BMAL1 heterodimer, an action that was shown to be specific to neurons (Yujnovsky et al., 2006). The D_2_ receptor also may regulate the rhythmic transcription of melanopsin mRNA in ipRGCs (Sakamoto et al., 2005), possibly influencing light entrainment signals being sent to the SCN. Additionally, DA appears to inhibit the activity of melatonin and aralkylamine *N*-acetyltransferase (AA-NAT, also known as serotonin *N*-acetyltransferase), a key enzyme in the production of melatonin (Iuvone, 1986; Cahill and Besharse, 1991). This inhibition was shown to be mediated through the D_2_ receptor, because D_2_ agonists, such as LY171555 and quinpirole, mimicked DA’s inhibition of AA-NAT activity (Iuvone, 1986) and melatonin release (Cahill and Besharse, 1991) and also mimicked the effects of light exposure (Iuvone, 1986; Cahill and Besharse, 1991).

Without retinal DA, cone-driven light adaptation (indicative of nighttime to daytime transition), contrast sensitivity, and visual acuity decrease (Jackson et al., 2012). Dopaminergic modulation of these retinal functions appears to be largely through the activity of D_1_ and D_4_ receptors (Jackson et al., 2012). Both receptors appear to increase the light-adapted amplitude of a b-wave (representative of ON-bipolar cell functionality); however, visual acuity appears to only be impacted by D_1_ receptors, and contrast sensitivity, by D_4_ receptors (Jackson et al., 2012; Hwang et al., 2013). Daytime contrast sensitivity, in turn, is also regulated by RGCs that express the clock protein NPAS2 (Hwang et al., 2013). Interestingly, DA, through activation of the D_4_ receptor, influences the proper expression of the *Npas2* gene, and regulates contrast sensitivity (Hwang et al., 2013). The activation of NPAS2 likely drives the activity of RGC’s AC1 (Hwang et al., 2013). Support for this hypothesis comes from the finding that expression of the D_4_ receptor gene, *D4d* (which fluctuates on a circadian rhythm similar to that of retinal DA), is necessary for the expression of *Adcy1*, a gene that produces AC1 (Jackson et al., 2011). This was confirmed with pharmacological manipulation, where the D_4_ antagonist, L745870, decreased the daytime expression of *Adcy1* (Jackson et al., 2011). Therefore, activation of the D_4_ receptor, during the daytime, is required for rhythmic fluctuation of cAMP through the regulation of AC1, and thus, contrast sensitivity and other downstream effects of the second messenger system (Jackson et al., 2011, 2012; Hwang et al., 2013).

Recent additional evidence also suggests that D_4_ receptors are involved in light adaptation (Prigge et al., 2016). Melanopsin-containing M1 ipRGCs, involved in sending light information for circadian photoentrainment (Gooley et al., 2001; Hattar et al., 2006), have been shown to presynaptically activate dopaminergic amacrine cells (Prigge et al., 2016). Mice without viable M1 ipRGCs have impaired light adaptation, which was rescued by the D_4_ receptor agonist, PD168077 (Prigge et al., 2016). D_4_ receptors expressed on cone photoreceptors (Witkovsky, 2004) allow for proper presynaptic feedback from active M1 ipRGCs and DA amacrine cells and optimal light adaptation (Prigge et al., 2016).

### Dopamine’s Modulation of Retinal Gap Junction Channels

The retina is rich in gap junctions, which contribute to different retinal functions by coupling (opening) and uncoupling (closing) to allow and block fast current transmission between retinal neurons. Some of these functions may be to set the period of retinal oscillators (Jaeger et al., 2015). Layers of the mammalian retina *in vitro* were found to have different oscillators, which oscillate on a 26-h period (Jaeger et al., 2015). This independent oscillation was shown to be due to gap junction coupling between the retinal layers (Jaeger et al., 2015). One of the layers that expresses an independent period is the inner nuclear layer (Jaeger et al., 2015), which contains the dopaminergic amacrine cells.

Retinal AII amacrine cells, which are important for rod photoreceptor-mediated vision, adapt to light environments by communicating with neighboring AII amacrine cells through the gap junction channel Cx36 (Kothmann et al., 2009). The uncoupling mechanism of retinal Cx36 channels is modulated by DA, released from neighboring amacrine cells (Kothmann et al., 2009). This effect is mediated by DA’s action on the D_1_ receptor, which activates the following intracellular mechanism: D_1_ receptor’s Gα_S_ protein activates AC and PKA, which phosphorylates protein phosphodiesterase 2 A, which dephosphorylates the serine 293 residue of the Cx36 channel, effectively uncoupling and shutting the channel (Kothmann et al., 2009). This mechanism blocks off communication between neighboring amacrine AII cells (Kothmann et al., 2009). Since DA uncouples amacrine cells’ Cx36 gap junctions by activating the above-described D_1_ receptor-driven mechanism (Kothmann et al., 2009), it may be that another D_1_ receptor-driven mechanism could set the phase of PER2 in amacrine and other retinal cells (Ruan et al., 2008; Jaeger et al., 2015).

In the fish retina, horizontal H1 cells are also coupled via gap junction channels, which the D_1_ receptor also uncouples during the day (Ribelayga and Mangel, 2003). An interesting observation from this study was that these gap junctions were coupled to each other in constant darkness (during the subjective day and subjective night) (Ribelayga and Mangel, 2003). This implies that the circadian-driven DA release was not enough to uncouple these gap junctions, because either photopic light or the D_1_ receptor agonist SKF38393 was required to significantly uncouple these gap junctions (Ribelayga and Mangel, 2003). This may be due to the fact that, unlike D_2_-like receptors, D_1_ receptors are less sensitive to DA, and the DA release during the circadian subjective day was too low to surpass the threshold for D_1_ activation and gap junction uncoupling (Ribelayga and Mangel, 2003). However, because D_2_-like receptors are more sensitive to DA, then the circadian-driven DA release could be enough to regulate gap junctions of other cells (Ribelayga and Mangel, 2003).

In the photoreceptor layer, the D_2_-like D_4_ receptors regulate the coupling of gap junctions between rod and cone photoreceptors (Ribelayga et al., 2008; Li et al., 2013; Jin et al., 2015; Zhang et al., 2015). Unlike the fish horizontal H1 cells, the gap junctions between the fish photoreceptors were able to couple during the subjective night and uncouple during the subjective day (Ribelayga et al., 2008). The uncoupling of these gap junctions is mediated by D_2_-like receptor activation, both in fish and mouse retinas (Ribelayga et al., 2008). Increased nighttime coupling (when DA levels are low) would, therefore, increase rod-driven dim light transmission through other rods and cones (Ribelayga et al., 2008). This nighttime transmission would increase the signal-to-noise ratio of transduction of dim light, but coupled rods’ response to single-photons would be decreased (Jin et al., 2015). It has been confirmed that the gap junction channel between these photoreceptors is Cx36, mediated by D_4_ receptors, which uncouple Cx36 through inhibition of the AC/PKA pathway (Li et al., 2013). Interestingly, adenosine and its receptor, A2aR, which also have rhythmic expression, appear to increase the nighttime coupling of photoreceptor Cx36 by activating the AC/PKA pathway (Li et al., 2013). Finally, it was confirmed that Cx36 channel phosphorylation (leading to coupling) fluctuates on a circadian scale and that photopic light decreases Cx36 phosphorylation (Zhang et al., 2015). These studies show that DA influences the gap junction uncoupling through several layers of the retina, both in fish and mice. Uncoupling of the photoreceptor gap junctions via D_4_ may influence the contrast sensitivity of the retina, while uncoupling through D_1_ receptors may influence the visual acuity (Jackson et al., 2012).

### The Co-release/Co-transmission of DA and GABA in the Retina

Retinal amacrine cells have been shown to release both DA and γ-amino butyric acid (GABA) (Hirasawa et al., 2009), although it is not clear whether these two neurotransmitters are co-released (same synaptic vesicle) or co-transmitted (same neuron, but different synaptic vesicles) (Vaaga et al., 2014). GABA proves to be important in the regulation of retinal circadian rhythms, because it decreases the amplitude of PER2 levels in the retina in a dose-dependent manner (Ruan et al., 2008). Because retinal GABA is an important neurotransmitter in modulating the clock proteins of the retina (Ruan et al., 2008), then GABA release by dopaminergic neurons in the OB (Maher and Westbrook, 2008; Kosaka and Kosaka, 2009; Kiyokage et al., 2010; Liu et al., 2013), the midbrain (Tritsch et al., 2012; Chuhma et al., 2014), and the hypothalamus (Zhang and van den Pol, 2015) may also be important in the modulation of circadian-like activities.

### Parkinson’s Disease and the Retina

Patients with PD have a number of types of visual dysfunction, some which may relate to altered retinal DA levels (see Archibald et al., 2009). Studies have shown that PD patients have thinner inner retinal layers, where endogenous dopaminergic amacrine cells reside (specifically, in the inner plexiform layer) (Witkovsky, 2004; Hajee et al., 2009; Adam et al., 2013; Spund et al., 2013). Disruptions of retinal dopaminergic neurons resulting in reduced retinal DA levels (Nguyen-Legros, 1988; Harnois and Di Paolo, 1990) may contribute to visual symptoms such as impaired foveal vision (Bodis-Wollner, 2009), contrast sensitivity (Bulens et al., 1989; Popova, 2014), and light adaption (Prigge et al., 2016) in patients with PD (Archibald et al., 2009). Impaired color and contrast discrimination may be a preclinical signs of PD (Chaudhuri and Schapira, 2009).

Decreased retinal DA levels in PD patients also have potential implications to circadian function. In mammals, photic entrainment of circadian rhythms occurs when ipRGCs convey photic signals to the SCN via the retinohypothalamic pathway (Gooley et al., 2001; Hattar et al., 2006). DA has been shown to regulate rhythmic melanopsin mRNA expression of these ipRGCs, possibly via actions at D_2_ receptors (Sakamoto et al., 2005). Dopaminergic amacrine cells in the retina also express circadian rhythms in expression of clock genes (Dorenbos et al., 2007).

## Olfactory Bulb

The first region of the nervous system to receive and process odorant information is the mammalian OB (see Figure 11 in Kosaka and Kosaka, 2016). Chemical odorants activate OSNs in the olfactory epithelium, which transmit odor signals to neurons in the GL of the OB. Synaptic contact between the OSNs and OB neurons occurs in discreet spherical structures known as glomeruli, where OSNs form glutamatergic axodendritic synapses with the main OB output neurons, mitral and tufted cells (M/TCs) (Berkowicz et al., 1994; Ennis et al., 1996), as well as local interneurons (Ennis et al., 2001). Surrounding the glomeruli, three morphologically distinct populations of interneurons exist: PGCs, SACs, and ETCs (Golgi, 1875; Shepherd, 1972; Shepherd et al., 2011; Nagayama et al., 2014). Collectively, these interneurons are known as JGCs. In addition to having synaptic contacts with each other, JGCs target both OSN terminals and M/TCs (Shepherd, 1972; Hsia et al., 1999; Berkowicz and Trombley, 2000; Ennis et al., 2001; Davila et al., 2003; Nagayama et al., 2014). Dopaminergic OB neurons are JGCs that are found in the GL (Baker, 1986; Ennis et al., 2001; Kosaka and Kosaka, 2009). An estimated 11% (∼87,000 neurons) of JGCs in the mouse OB are dopaminergic (Parrish-Aungst et al., 2007), while the adult rat has an estimated 100,000–150,000 dopaminergic neurons in the GL (McLean and Shipley, 1988). DA receptors are found throughout the entire OB, with D_1_ receptors localizing in all layers but the ONL, and D_2_ receptors heavily localizing in the GL and ONL (Nickell et al., 1991; Hsia et al., 1999; Berkowicz and Trombley, 2000; Ennis et al., 2001; Davila et al., 2003).

As in the retina, the OB has been shown to have circadian rhythms that are independent of the master circadian pacemaker, the SCN, allowing the OB to maintain its daily rhythmicity *in vitro* (Granados-Fuentes et al., 2004a,b). However, the neuronal mechanisms that drive the independent circadian activity of the OB are not well known. DA, which has a daily rhythm of release in the rat OB (Corthell et al., 2013), may be involved in modulating the circadian activity of the OB via a variety of mechanisms (and DA itself may be regulated through circadian mechanisms) (Mendoza and Challet, 2014).

### Daily Dopamine Activity in the Olfactory Bulb

In the rat OB, DA content rhythmically fluctuates depending on the light cycle (Corthell et al., 2013). The overall activity was determined by measuring the ratio of DA’s metabolite, DOPAC (representative of DA release), to DA every 2.5 ZT hour (Corthell et al., 2013). DOPAC/DA ratios were shown to be highest during ZT 0-12 (lights turned on) and lowest during ZT 13-24 (lights turned off) (Corthell et al., 2013). Since DA release follows a diurnal variation, it may be that circadian activity in the OB also occurs in dopaminergic neurons (Mendoza and Challet, 2014).

### Dopamine’s Presynaptic Inhibition of Olfactory Sensory Neurons

An abundance of electrophysiological data has shown that DA, released by JGCs, presynaptically inhibits transmission between OSNs and OB neurons by activating D_2_ receptors on OSN terminals (Hsia et al., 1999; Berkowicz and Trombley, 2000; Ennis et al., 2001). A subsequent study by our group showed that DA also inhibits excitatory transmission between M/TCs and interneurons by a presynaptic mechanism involving D_2_ receptors (Davila et al., 2003). In addition, DA also induces excitation in postsynaptic interneurons via the D_1_ receptor (Liu et al., 2013). Through these actions, and the diurnal release of DA, it is hypothesized that DA sharpens synaptic processing of olfactory information by diminishing tonic odorant noise (Berkowicz and Trombley, 2000; Ennis et al., 2001).

Since OSNs and M/TCs excite their postsynaptic targets via glutamate release, DA’s presynaptic inhibition decreases the release of glutamate onto postsynaptic targets, effectively keeping them from becoming excited and transmitting the odor signals to higher brain regions (Hsia et al., 1999; Berkowicz and Trombley, 2000; Ennis et al., 2001). Inhibition by DA appears to be presynaptic, because application of exogenous DA did not change the kinetics of the postsynaptic response of M/TCs to olfactory nerve stimulation, indicating that both NMDA and AMPA/kainate receptor-mediated components were affected equally (Berkowicz and Trombley, 2000). Also, DA did not directly affect currents evoked by exogenous application of glutamate to cultured OB neurons (Berkowicz and Trombley, 2000; Davila et al., 2003). Further supporting a presynaptic mechanism, DA altered the ratio between the conditioning and the test response amplitude recorded in postsynaptic neurons in rat OB slices, thereby attenuating the degree of paired-pulse depression (Ennis et al., 2001).

### Dopamine May Act as a High-Pass Filter for Olfactory Bulb Signal Transduction

All neurons that store and release chemical neurotransmitters via vesicular exocytosis have the potential to deplete their vesicular storage with a high-frequency stimulus. Conversely, neurons may not release any vesicles if the signal is too low in frequency. Most neurons are gated to a specific frequency that would allow for vesicular exocytosis. Therefore, neurotransmitters that are released by low-frequency, but not high-frequency, stimuli are regulated by a low-pass filter, while neurotransmitters that are released by high-frequency, but not low-frequency, stimuli are regulated by a high-pass filter. The release of DA in the OB may be gated by a low frequency, suggesting that a low-pass filter is needed for DA release. However, DA itself may influence OB signal transduction as a high-pass filter.

Theta frequency (4–10 Hz), which is observed in the OB’s GL (Fukunaga et al., 2014), may be needed to release DA and other neurotransmitters. Theta frequency is also implied to be the sniffing frequency at which rodents sample odors (Wachowiak, 2011; Liu et al., 2013; Genovese et al., 2016), and this low frequency could also increase the signal-to-noise ratio. Therefore, if OB DA is released while sniffing at a theta frequency, DA release may inhibit low, tonic odor transmission. If a nocturnal animal is sampling odors while awake (when OB DA content is low), or while asleep during the day (when OB DA content is high), potent odors may activate dopaminergic interneurons so frequently that eventually the vesicular DA storage will be depleted. Additionally, constant DA release and binding can desensitize the presynaptic D_2_ receptors on OSNs (Beaulieu et al., 2007; Clayton et al., 2014). Constant D_2_ receptor activation would lead to phosphorylation of these receptors by G-protein receptor kinases, which would recruit the arrestin proteins, eventually leading to an internalization of the receptor (Beaulieu et al., 2007; Clayton et al., 2014). Thus, DA release may be gated by a low-pass filter, but DA activity, itself, may act as a high-pass filter, allowing for only those odors that surpass a threshold to be transmitted.

### Dopamine and GABA Co-release in the Olfactory Bulb

In the OB, nearly 97% of all DA JGCs co-express glutamate decarboxylase 67, the rate-limiting enzyme in GABA production (Kiyokage et al., 2010), indicating that GABA is released from DA cells (Maher and Westbrook, 2008; Kosaka and Kosaka, 2009; Borisovska et al., 2013; Liu et al., 2013). It is interesting to note that even though there are species differences between the chemical composition of rat and mouse OB JGCs, the dopaminergic neurons of both species largely co-express GABA (Kosaka and Kosaka, 2007, 2016). One of the JGCs that co-expresses DA and GABA is the SAC (Hökfelt et al., 1975; Kosaka and Kosaka, 2008; Kiyokage et al., 2010; Liu et al., 2013). Evidence suggests that SACs release both GABA (inhibition through GABA_A_ receptor) and DA (excitation through D_1_ receptor) onto neighboring and distant glomeruli, generating a biphasic inhibition-excitation response (Liu et al., 2013).

The DA-GABA co-release mechanism may be linked to a rodent’s sampling rate of novel odors (Liu et al., 2013). SACs form synapses with ETCs, which release glutamate onto all other OB interneurons and the main output cells, M/TCs (Liu et al., 2013; Nagayama et al., 2014). GABA and DA, co-released from SACs, lead to a temporal, biphasic inhibition-excitation response in ETCs (Liu et al., 2013), which facilitates or dampens the transmission of olfactory signals to higher brain regions (Liu et al., 2013). A feed-forward inhibition of M/TCs also would occur when some ETCs activate GABAergic PGCs (Liu et al., 2013). These GABA and DA actions may indirectly allow some M/TCs to send odor signals, while other M/TCs are briefly inhibited. Therefore, a rodent may process important odors (e.g., predators, food) more selectively while it is sleeping during the day, which is when OB DA activity is reported to be highest (Corthell et al., 2013).

### Biophysical Properties and Daily Expression of the Connexin 36 in the Olfactory Bulb

As in the retina, Cx36 gap junction channels are expressed in the OB (Belluardo et al., 2000; Christie and Westbrook, 2006) and play a crucial role in the transmission of the odorant signals in the OB within the mitral cells (Christie and Westbrook, 2006). Specifically, mitral cell dendrites couple to each other in their respective glomeruli, allowing for lateral excitation (Christie and Westbrook, 2006). Cx36 channels help to amplify an odorant signal sent from the sensory neurons onto the mitral cells by having depolarization spread from one mitral cell to the other neurons coupled to it (Christie and Westbrook, 2006). Cx36 channels were confirmed to be involved in mitral cell lateral excitation, because Cx36 knock-out mice did not show lateral excitation of mitral cells within the same glomerulus (Christie and Westbrook, 2006).

Cx36 mRNA has a daily expression rhythm in the OB of the rat, with highest expression during nighttime and lowest expression during daytime (Corthell et al., 2012), out of phase with DA release (Corthell et al., 2013). This may imply that Cx36 gap junctions also play a role in the diurnal rhythm of olfactory acuity in rodents (Granados-Fuentes et al., 2006, 2011). And, if DA plays a role in uncoupling Cx36 channels of the mitral cells, as it does in inhibiting glutamate release from mitral cells (Davila et al., 2003), then DA may further dampen an odor signal being sent from a mitral cell by inhibiting lateral excitation among mitral cells.

### The Olfactory Bulb Maintains its Own Daily Rhythms

Increasing evidence suggests that the OB may function as an independent circadian system controlling daily changes in olfaction (Miller et al., 2014). For example, the OB exhibits intrinsic circadian rhythms in firing rate and clock gene (*Per1*) activity *in vitro* (Granados-Fuentes et al., 2004b). Interestingly, daily rhythms in the OB and olfactory discrimination persist when circadian rhythms are eliminated in the SCN (Abraham et al., 2005) or when the SCN is ablated (Granados-Fuentes et al., 2004a). A later study from this group showed that daily rhythms in olfactory discrimination in mice depend on clock gene expression but not the SCN (Granados-Fuentes et al., 2011), while the SCN is still necessary for entrainment of the OB in the intact animal (Granados-Fuentes et al., 2004a). More recently, this group showed that VIP regulates circadian rhythms in gene expression and odor detection performance in the OB (Miller et al., 2014). Data from our lab suggesting diurnal variations in DA release in the OB (Corthell et al., 2013) raise the possibility of circadian activity of dopaminergic cells in the OB as well (Mendoza and Challet, 2014).

### Dopamine in the Human Olfactory Bulb

Dopamine’s presynaptic mechanism of inhibiting glutamate release from the OSNs in turtles and mice (Berkowicz and Trombley, 2000; Ennis et al., 2001) also may be present in the human OB, since human OBs have been shown to have dopaminergic neurons in the GL (Alizadeh et al., 2015). Interestingly, when labeling postmortem human brains with the TH antibody, it was shown that younger (35 years and under) postmortem OBs had less TH-positive cells than older (50 years and over) postmortem OBs (Alizadeh et al., 2015). Rodents also show an increase in OB dopaminergic neurons with age (Kosaka and Kosaka, 2009). This increase in dopaminergic neurons in older OBs may be caused by the progression of PD, as discussed below.

Alizadeh et al. (2015) have proposed that younger people tend to detect odors better than older people (confirmed previously by Doty et al., 1984), because the over suppression of olfactory information processing by DA presynaptically inhibits the release of glutamate from OSNs onto mitral cells in older people (Doty and Risser, 1989; Alizadeh et al., 2015). Although this presynaptic DA action is proposed to sharpen odor detection by filtering noise (Berkowicz and Trombley, 2000; Ennis et al., 2001), an increase in DA OB neurons may suggest excessive DA activity. Based on the previous cited evidence (Ennis et al., 2001; Davila et al., 2003), excessive DA activity could lead to a saturation of D_2_ receptor activation, which would not only block odor signal transmission by inhibiting glutamate release, but also by potentially uncoupling Cx36 channels between coupled mitral cell dendrites.

### Parkinson’s Disease and the Olfactory Bulb

Patients with PD often have types of olfactory dysfunction (Doty et al., 1988), which can be an early precursor to the development of PD (Ross et al., 2008). Close relatives of patients with idiopathic PD are much likelier to develop idiopathic PD and bradykinesia if they have hyposmia (a diminished sense of smell) but not normosmia (normal sense of smell) (Berendse et al., 2001; Ponsen et al., 2004). These and other clinical studies provide evidence that non-motor smell tests are superior diagnostic tools for PD to motor tests, especially in people with mild PD who have yet to develop bradykinesia (Bohnen et al., 2008). Non-motor smell tests may serve as predictors of those who will develop PD (Berendse et al., 2001; Ponsen et al., 2004).

How are the endogenous DA neurons of the OB affected by PD? Paradoxically, unlike the midbrain and, potentially, the retina, DA and TH neurons *increase* in the OBs of patients with PD (Huisman et al., 2004; Mundinano et al., 2011). An increase in dopaminergic OB neurons has also been shown in the transgenic rat line, α-synuclein, which exhibit signs of olfactory dysfunction that precede signs of motor dysfunction (Lelan et al., 2011). An increase in DA in the OB can contribute to hyposmia of PD patients (Huisman et al., 2004) by increasing DA binding to D_2_ receptors located on OSNs and M/TCs, causing a presynaptic inhibition of glutamate release from the OSNs and M/TCs, effectively inhibiting odor performance (Hsia et al., 1999; Berkowicz and Trombley, 2000; Ennis et al., 2001; Davila et al., 2003). This hypothesis is supported by a study in which administration of the D_2_ agonist, quinpirole, caused a dose-dependent decrease in odor detection and performance in rats (Doty and Risser, 1989). Knockout mice lacking the dopamine transporter or D_2_ receptors have also been shown to have an odor discrimination deficit (Tillerson et al., 2006).

## Midbrain’s Substantia Nigra Pars Compacta and Ventral Tegmental Area

The striatum (a collective term for the caudate nucleus, NAcc, and the putamen of the basal ganglia) receives a large input from the SNc’s dopaminergic neurons through the nigrostriatal pathway. This dopaminergic input results in the facilitation of movement through direct (D_1_ receptor-driven) and indirect (D_2_ receptor-driven) pathways (see Figure 1 in Nelson and Kreitzer, 2014). In addition to D_1_ and D_2_ receptors, the D_3_ receptor is expressed in the striatum (most heavily in the NAcc), the SN, and VTA (see Beaulieu and Gainetdinov, 2011). The D_4_ and D_5_ receptors also show minimal expression in the SN (see Beaulieu and Gainetdinov, 2011). The D_2_ receptor pathway appears to be especially important for driving the expression the clock protein PER2 in the dorsal striatum (Hood et al., 2010). Therefore, DA may be important in modulating circadian activities of the striatum, including daily locomotion, something that is lost in patients with PD.

The midbrain also contains the VTA, from which DA neurons project to different brain areas, including cortical (mesocortical projections) and limbic (mesolimbic projections) areas (Russo and Nestler, 2013). Dopaminergic neurons in the mesolimbic pathway (often called the reward pathway, see Figure 1 in Russo and Nestler, 2013) project to the hippocampus, NAcc, and the amygdala, are important for learning and motivation, and are well known to be influenced by drugs of abuse. In fact, prolonged exposure to cocaine in mice increases the expression of *Per1* and *Clock* mRNA, and it decreases the expression of *Per2*, *Bmal1*, *Cry1*, and *NPAS2* (a paralog of *Clock*) mRNA in the striatum (Uz et al., 2005). Cocaine may lead to addictive behaviors by altering the expression of circadian genes in the striatum (Uz et al., 2005) by increasing DA activity from the midbrain.

### Dopamine Receptors’ Effects on Clock Gene and Protein Expression in the Striatum

The striatum receives a large dopaminergic input, and so it is not surprising that clock genes and proteins have various interactions with DA. Cultured mouse striatal neurons have been shown to express genes for DA receptors 1, 2, and 3, as well as for clock genes *Per1*, *Clock*, *Bmal1*, and *NPAS2* (Imbesi et al., 2009). These DA receptors differently regulate the expression of these clock genes. Application of the D_1_ receptor agonist, SKF-38393, increased mRNA levels of all of the above-mentioned clock genes, while the D_2_ receptor agonist, quinpirole, decreased *Clock* and *Per1* mRNA levels (Imbesi et al., 2009). Additionally, quinpirole injections were shown to increase nighttime expression of PER1, but decrease the protein’s daytime expression (Imbesi et al., 2009). The following 2010 study by Hood et al. (2010) shows that the D_2_ receptor is necessary for proper PER2 expression.

In the rat’s dorsal striatum, the expression of PER2 peaks during the daytime hours, while extracellular DA peaks during the nighttime hours (Hood et al., 2010). Lesioning DA neurons via a unilateral injection of 6-hydroxydopamine significantly decreased the ipsilateral dorsal striatum’s PER2 protein and mRNA expression 14 days after the injection, without evidence of restoration (Hood et al., 2010). Injections of α-methyl-*para*-tyrosine (AMPT), an inhibitor of TH, decreased DA content and daytime (ZT 1) PER2 expression, but increased PER2 expression during nighttime (ZT 13) (Hood et al., 2010). Lastly, the D_2_ receptor antagonist, raclopride, injected chronically, decreased PER2 expression at ZT 1, while the agonist quinpirole restored and reversed the daily PER2 expression in rats previously treated with 6-hydroxydopamine on the lesioned side (Hood et al., 2010).

### Daily Tyrosine Hydroxylase and Dopamine Activity in the Midbrain

The expression of VTA’s TH and the firing of dopaminergic neurons are both linked to a specific time of the day (Webb et al., 2009; Chung et al., 2014; Dominguez-Lopez et al., 2014; Sidor et al., 2015). Interestingly, the diurnal variation in VTA’s TH protein expression appears to out-of-phase with the *TH* mRNA expression (Sidor et al., 2015). TH in the VTA is expressed more during the daytime (around ZT 6) than during the nighttime (ZT 13-24), while *TH* mRNA is expressed less during the daytime (ZT 4) than the nighttime (ZT 16, 20) (Webb et al., 2009; Sidor et al., 2015). Circadian expression of *TH* mRNA in the VTA is also low during the subjective day (CT 8 and 12) and high during the subjective night (CT 20 and 24) (Chung et al., 2014). Additionally, TH expression appears to be similar in the VTA and SNc, where both show peak expression at CT 0 and lowest expression at CT 12 (Chung et al., 2014). However, TH expression in the VTA and NAcc is out-of-phase, with both the peak for VTA TH expression and the trough for the NAcc TH expression occurring at ZT6 (Webb et al., 2009).

Tyrosine hydroxylase expression in the VTA is suppressed by CLOCK (McClung et al., 2005; Sidor et al., 2015) and by the circadian nuclear receptor REV-ERBα (Chung et al., 2014). Homozygous *Clock* mouse mutants have an increased expression of TH in their VTA (McClung et al., 2005). Higher VTA expression of TH would cause an increase in DA production and release in the mesolimbic pathway, likely being one of the causes for the reported results of *Clock* mutant mice finding cocaine more rewarding and expressing higher sensitization to cocaine (McClung et al., 2005). *Rev-erb*α knock-out mice exhibited increased *TH* mRNA expression and DA release at CT 12 (Chung et al., 2014). Increased TH and DA through loss of REV-ERBα led to increased mania-like behavior in these mice (Chung et al., 2014).

Monoamine oxidase A (MAOA), an enzyme that suppresses DA activity by removing DA’s amine group, is also controlled by circadian mechanisms. Midbrain’s MAOA function is highly important in regulating DA activity and, therefore, mood and movement. MAOA and its mouse gene variant, *Maoa*, were found to have rhythmic expression in the VTA, with highest expression at ZT 6 and lowest at ZT 18 (Hampp et al., 2008). Conversely, *Per2* mutant mice had a flat daily expression of *Maoa* and MAOA activity, which unsurprisingly led to higher levels of DA release and activity (Hampp et al., 2008). *Per2* mutants also experienced less helplessness in a forced swim test compared to control mice, likely a result of increased DA due to decreased MAOA (Hampp et al., 2008). The daily rhythmic expression of TH and MAOA may be a mechanism of daily mood fluctuation in people.

The firing activity of VTA neurons, as electrophysiology data show, does not appear to be consistent throughout studies (Chung et al., 2014; Dominguez-Lopez et al., 2014; Sidor et al., 2015). *In vivo* recordings from DA neurons in the VTA showed that these neurons fire the most spikes during the onset of daytime (7:00–11:00 h) and onset of nighttime (19:00–23:00 h), while firing significantly less spikes in between (11:00–15:00 h and 23:00–3:00 h) (Dominguez-Lopez et al., 2014). Therefore, a 12-h period for the spike firing frequency is shown in these VTA neurons (Dominguez-Lopez et al., 2014). However, other *in vivo* recording data show a steady firing rate of VTA neurons (Chung et al., 2014; Sidor et al., 2015). This discrepancy may be due to inherent species difference, since the twice-daily highest spiking frequency was found in rats (Dominguez-Lopez et al., 2014) and the steady firing frequency was recorded in mice (Chung et al., 2014; Sidor et al., 2015). It may not be surprising that both CLOCK and REV-ERBα suppress VTA firing, as they also suppress TH expression (McClung et al., 2005; Chung et al., 2014; Sidor et al., 2015). Mutant mice without a functional *Clock* have higher bursts and firing rates (McClung et al., 2005; Sidor et al., 2015), while an antagonist of REV-ERBα, SR8278, increased the firing rate of VTA neurons equally during two different time points (ZT 1 and 11) (Chung et al., 2014).

### Dopamine’s Presynaptic Inhibition of Glutamate Release onto the Ventral Tegmental Area

Similar to the effect of DA on olfactory-nerve-evoked responses in OB neurons (Hsia et al., 1999; Berkowicz and Trombley, 2000; Ennis et al., 2001), VTA’s DA inhibits glutamate release presynaptically via the D_2_ receptor (Koga and Momiyama, 2000). Focal stimulation within the VTA in the presence of various pharmacological blockers evoked non-NMDA EPSCs in dopaminergic neurons (Koga and Momiyama, 2000). Addition of exogenous DA inhibited these EPSCs (Koga and Momiyama, 2000). Repeating these experiments with the D_2_ receptor agonist, quinpirole, produced similar results in both the VTA and OB, indicating that this inhibition is specific to D_2_ receptors (Hsia et al., 1999; Berkowicz and Trombley, 2000; Koga and Momiyama, 2000; Ennis et al., 2001). DA also reduced the frequency of spontaneous miniature EPSCs in VTA neurons without affecting their mean amplitude, suggesting that the inhibitory effects of DA are presynaptic (Koga and Momiyama, 2000).

### Dopamine and GABA Co-release from Midbrain Dopaminergic Neurons

Midbrain VTA and SNc DA neurons provide input onto the striatum that is not strictly dopaminergic. Midbrain DA neurons have been shown to co-release DA and GABA onto medium spiny neurons of the striatum (Tritsch et al., 2012; Borisovska et al., 2013; Chuhma et al., 2014; Vaaga et al., 2014). This co-release produces a large GABA_A_ current that inhibits medium spiny neurons (Tritsch et al., 2012). Interestingly, these dopaminergic neurons package GABA into vesicles via the vesicular monoamine transporter 2, a traditional DA transporter, and not by the vesicular GABA transporter (Tritsch et al., 2012).

Cholinergic interneurons in the NAcc core also receive a heterogeneous input from the midbrain (Chuhma et al., 2014). Those neurons receive dopaminergic and GABAergic input from the midbrain DA neurons, causing hyperpolarization via the D_2_ and GABA_A_ receptors, respectively (Chuhma et al., 2014). However, cholinergic interneurons in the NAcc medial shell do not receive GABAergic input from midbrain dopaminergic neurons (Chuhma et al., 2014). This heterogeneity of midbrain’s dopaminergic input to the NAcc and dorsal striatum could be involved with reward-related learning (Chuhma et al., 2014).

### Gap Junctions in the Midbrain’s Dopaminergic and GABAergic Neurons

As with all other dopaminergic brain areas discussed in this review, the midbrain is also rich in gap junctions (Stobbs et al., 2004; Vandecasteele et al., 2005; Allison et al., 2006). Within the VTA, GABA neurons are strongly coupled to each other via Cx36 gap junctions (Stobbs et al., 2004), with an estimate of 2,000 channels per cell (Allison et al., 2006). The presence of Cx36 was confirmed with quantitative reverse transcription polymerase chain reaction, immunohistochemistry, and by the reduction of spiking after the application of the Cx36 specific blocker, mefloquine (Allison et al., 2006). It was suggested that VTA GABA neurons, not VTA DA neurons, express Cx36 channels, due to the fact that GABA VTA neurons have a different electrophysiological profile from DA neurons (Allison et al., 2006). However, recent evidence may imply that these GABA VTA neurons also express DA, because it was shown that VTA, as well as SNc, projection neurons express both DA and GABA (Tritsch et al., 2012; Chuhma et al., 2014). In the SNc of rat pups, gap junctions are expressed differently during postnatal development (Vandecasteele et al., 2005). For example, it was found that 40% of neurons from rats aged between P5 and P10 were coupled, that no neurons were coupled between the ages of P10 and P15, and that 17% of neurons were coupled between the ages of P10 and P15 (Vandecasteele et al., 2005). It would be informative to test for Cx36 and vesicular monoamine transporter 2 immunoreactivity in the VTA to confirm whether VTA DA neurons also express Cx36 gap junctions and also to observe how gap junction coupling changes later in rodent development.

### Parkinson’s Disease and the Midbrain and Striatum

The degeneration of the dopaminergic neurons in the SNc is the most well-known cause of motor symptoms in PD, including bradykinesia (Carlsson, 1972). However, increasing evidence suggests that midbrain DA loss in patients with PD could lead to motor and non-motor circadian dysfunction. As mentioned in the section “Introduction,” there are a number of limitations to neurotoxin-based and transgenic animal models of PD (see Fifel and Cooper, 2014). One exception is the transgenic MitoPark Parkinsonian mouse, which has been shown to develop progressive cellular and motor alterations analogous to those seen in idiopathic PD, including a gradual loss of midbrain (e.g., VTA and SN) dopaminergic neurons (Ekstrand et al., 2007; Galter et al., 2010; Fifel and Cooper, 2014).

This MitoPark mouse was used to study the rest/wake activities and locomotion throughout the progression of PD in the mouse’s lifetime (Fifel and Cooper, 2014). To assess daily rest and locomotor activity, as well as how lighting could affect these activities, extensive entrainment periods of light and dark conditions were used (Fifel and Cooper, 2014). These entrainment periods lasted for several weeks and showed evidence of the age-related degeneration of dopaminergic neurons with progression of the disease (Fifel and Cooper, 2014).

Prior to the onset of PD, 11-week-old MitoPark mice entrained well to the 12-h:12-h light-dark cycle, and when exposed to a 1 h light pulse and then released into constant darkness, showed a phase delay of activity that was comparable to that in their control counterparts (Fifel and Cooper, 2014). However, by age 26 weeks, when DA levels in the nigrostriatal regions of the MitoPark mouse have been shown to be decreased (Galter et al., 2010), the locomotor activity of MitoPark mice had become completely arrhythmic when the mice were re-assessed under constant darkness (Fifel and Cooper, 2014). MitoPark mice also lost circadian control of locomotor activity when exposed to constant, bright light (Fifel and Cooper, 2014). However, over the experiment’s duration, the daily rhythm of rest/activity in a light-dark cycle in MitoPark mice was maintained (Fifel and Cooper, 2014).

In addition to motor symptoms, PD is associated with non-motor symptoms involving loss of midbrain DA. Patients with PD often experience dysfunction of REM sleep (Chaudhuri and Schapira, 2009), which appears to be regulated by dopaminergic neurons in the SNc (Lima et al., 2007). In an MPTP rat model of PD, a strong correlation was found between the number of SNc DA neurons lost and the percentage decrease in REM sleep (Lima et al., 2007). Patients with PD may develop RBD, which is characterized by the enacting of dreams, often with flailing of the limbs (Chaudhuri and Schapira, 2009; Videnovic and Golombek, 2013). RBD has been shown to be present in nearly 25–50% of people with PD, and it is often a precursor to PD (Chaudhuri and Schapira, 2009; Lima, 2013).

Degeneration of the dopaminergic neurons in the midbrain’s SNc in patients with PD is also important to the striatum, as SNc dopaminergic neurons project to the striatum via the nigrostriatal pathway. TH, DA, and its metabolites, DOPAC and HVA, show daily rhythms of expression in the striatum (Castañeda et al., 2004; Webb et al., 2009), where DA regulates rhythms of clock gene and protein expression (Imbesi et al., 2009; Hood et al., 2010; Gravotta et al., 2011). In patients with PD, reduced DA input to the striatum due to nigrostriatal degeneration could blunt daily rhythms of clock gene expression and contribute to circadian disruptions (Videnovic and Golombek, 2013; Verwey et al., 2016). IPT-SPECT imaging data suggest that reduced striatal dopamine transporters may contribute to the pathophysiology of RBD (Eisensehr et al., 2003). Another sleep disorder found in patients with PD is RLS, with a reported prevalence of 8–50% (Videnovic and Golombek, 2013). Some imaging (PET) data support the hypothesis that disturbed striatal dopamine transmission is involved in the pathophysiology of RLS (Turjanski et al., 1999).

## Hypothalamus

One site for DA’s unique, circadian-like neuromodulatory activities is within the arcuate nucleus of the hypothalamus (see Figure 4 in Freeman et al., 2000; Bertram et al., 2010). There, DA regulates the daily rhythm of PRL, a hormone best known for its involvement with milk production in mammals, from the lactotrophs of the anterior pituitary gland (Freeman et al., 2000; Bertram et al., 2010). An essential role of the PRL rhythm is the maintenance of the corpus luteum and its release of progesterone during pregnancy in rodents (Freeman et al., 2000; Bertram et al., 2010). Therefore, DA also plays a role in the regulation of reproductive mechanisms. There are several DA neuronal subpopulations in the hypothalamus, at least three of which contribute to the inhibition of PRL release: TIDA, tuberohypophyseal dopamine neurons, and periventricular hypophyseal dopamine neurons (Gerhold et al., 2001). Additional data from this lab suggest that clock gene expression, particularly *Per1* and *Per2*, within TIDA neurons may regulate the daily rhythmicity of DA release from these neurons in ovariectomized rats (Sellix and Freeman, 2003; Sellix et al., 2006). This section focuses on the potential of TIDA neurons to regulate the circadian rhythms of PRL release and potential disruptions to PRL release in patients with PD. In regard to DA receptors, the arcuate nucleus expresses D_1_ and D_2_ receptors (Romero-Fernandez et al., 2014), and potentially D_3_ receptors on TIDA neurons (Lin et al., 2000).

This section also discusses the role of DA in the SCN, the site of the master clock. Interestingly, in one study, TH-positive neurons were labeled within the SCN of neonatal hamsters (Strother et al., 1998). The SCN expresses D_1_ and D_5_ receptors (Rivkees and Lachowicz, 1997), where DA from other brain regions exerts feedback effects (Mendoza and Challet, 2014). Finally, this section reviews potential links between PD and the SCN, including mixed results of animal studies implicating SCN involvement.

### Dopamine from TIDA Neurons and the Regulation of Prolactin Release

The TIDA neurons of the hypothalamus’ arcuate nucleus display a rhythmic release of DA, which is out of phase with PRL release (Freeman et al., 2000; see Figure 2 in Bertram et al., 2010). In humans, PRL release is high in the nighttime and low in the daytime, and DA release drops before the release of PRL (Freeman et al., 2000; Bertram et al., 2010). Therefore, TIDA DA release inhibits pituitary lactotrophs, decreasing PRL release (Freeman et al., 2000; Bertram et al., 2010). However, PRL also stimulates the activity of TIDA neurons, thereby increasing DA release (Freeman et al., 2000; Bertram et al., 2010). These feedback mechanisms can result from artificial cervical stimulation, which results in PRL being released twice per day, at 300 and 1700 h, and likely later in the day, while DA is released at around 1200 h (Freeman et al., 2000; Bertram et al., 2010). This twice-daily and daily release of PRL and DA, respectively, continues for a few days without additional cervical stimulation, potentially showing evidence of a circadian-like neuronal “memory” that drives the expression of PRL days after stimulation (Freeman et al., 2000; Helena et al., 2009; Bertram et al., 2010). It is thought that this memory regulates the rhythms of DA and PRL (Freeman et al., 2000; Helena et al., 2009; Bertram et al., 2010).

### GABA release from Hypothalamic Tuberoinfundibular Dopamine Neurons

Amongst other dopaminergic neuronal populations of the arcuate nucleus, TIDA neurons appear to also release GABA (Zhang and van den Pol, 2015). GABA release from the arcuate nucleus may inhibit PRL release (McCann and Rettori, 1986) and other neurons (including TIDA) of the arcuate nucleus, as well (Zhang and van den Pol, 2015). By using optogenetics, Zhang and van den Pol (2015) activated DA neurons of the arcuate nucleus while recording from other arcuate neurons, both TIDA and non-TIDA neurons. Recordings showed a large-amplitude, inward (depolarizing) current with fast decay, followed by a small-amplitude, outward (hyperpolarizing) current with slow decay (Zhang and van den Pol, 2015). This outward current was determined to be a GABA_A_ current, since it was blocked by the GABA_A_ antagonist bicuculline (Zhang and van den Pol, 2015). Therefore, TIDA neurons also release GABA, which hyperpolarizes neurons in the arcuate nucleus, and, potentially, the lactotrophs of the anterior pituitary (McCann and Rettori, 1986; Zhang and van den Pol, 2015). GABA release from TIDA neurons onto other arcuate nucleus neurons may help to keep hypothalamic DA activity low until 1200 h in cervically stimulated rats (Helena et al., 2009), and it may contribute to the circadian rhythmic activity in the hypothalamus.

### SCN and Role of Dopamine

The SCN, located above the optic chiasm, maintains mammalian circadian rhythms by being entrained by light it receives through the retinohypothalamic tract and by entraining the rhythms of peripheral regions. Within neurons of both the SCN and periphery, 24-h oscillations are generated through a clock gene transcriptional and translational feedback loop (Lowrey and Takahashi, 2011). DA is relevant to the SCN, as the main SCN clock communicates timing information with other brain clocks to regulate DA activity, and DA also appears to have feedback effects on the SCN (Mendoza and Challet, 2014).

A role for dopaminergic modulation of SCN-related circadian rhythmicity is suggested by the presence of D_1_ and D_5_ receptors in the SCN (Rivkees and Lachowicz, 1997). In fact, DA may be necessary to entrain the developing, fetal SCN (Mendoza and Challet, 2014). Prenatal exposure to the D_1_ receptor agonist, SKF38393, disrupts the expression of c-fos (a marker of neuronal activity) in the neonatal SCN (Ferguson et al., 2000). Light pulses also mimic the effects of D_1_ receptor activation in neonatal hamsters, suggesting that maternal DA represents daytime to the fetal SCN (Viswanathan and Davis, 1997).

Recent evidence that methylphenidate alters the electrical firing rate and clock gene expression in the SCN (Antle et al., 2012; Baird et al., 2013; Mendoza and Challet, 2014) suggests that some drug-induced changes in DA neurotransmission also may influence SCN clock activity. Data from mostly animal models of PD (see below), implicating dopamine in SCN dysfunction, are mixed, but suggest that DA depletion may affect circadian rhythm mechanisms differently in the SCN than in peripheral hypothalamic areas.

### Parkinson’s Disease and the Hypothalamus

The proposed idea that PD is disruptive to circadian rhythms would be central to the functioning of the hypothalamus, where the SCN resides. Mechanisms underlying circadian fluctuations in signs and symptoms in patients with PD remain unclear, although altered DA metabolism and DA receptor downregulation have been implicated (Videnovic and Golombek, 2013). Sites of disruption may be along afferent pathways to the SCN (e.g., impaired light transmission due to dopaminergic retinal degeneration), within the SCN itself (e.g., altered clock gene expression), or within downstream peripheral efferent pathways (e.g., altered output) (Videnovic and Golombek, 2013).

Data from mostly animal models implicating SCN dysfunction in PD is mixed. Clock gene and protein expression in the SCN is unaffected in some animal models of PD, but is affected in others. For example, studies have shown that the photoresponse of the SCN is unaltered in D_2_ receptor knockout mice, but masking (which could hide potential SCN dysfunction by exposing a nocturnal animal to light) was abolished in these mice (Doi et al., 2006). The expression of *Per2* and PER2 in the SCN is unaffected in some Parkinsonian animal models (Gravotta et al., 2011; Kudo et al., 2011). In addition, the rhythmic release of melatonin and cortisol (a function regulated by the SCN) is unaffected in Parkinsonian non-human primates (Fifel and Cooper, 2014; Fifel et al., 2014), which mirrors a finding that nighttime melatonin release in PD patients was similar to that of control patients (Fertl et al., 1993). While these findings suggest that the central rhythms of the SCN are unaffected in some models and patients of PD, other studies may contradict this notion.

The SCN of a Parkinsonian rat model (symptoms were induced by rotenone administration) displayed increased levels of expression of *Per2* at some time points, varying levels of expression of *Cry2* at different time points, and reduced levels of expression of *Per1* and *Bmal1* at some time points (Mattam and Jagota, 2015). In addition, the daily pulse of *Per1*, *Per2*, *Cry1*, *Cry2*, and *Bmal1* was significantly decreased in the SCN of the rotenone-induced PD rat model (Mattam and Jagota, 2015). In a Parkinsonian mouse model (initiated by the administration of MPTP), the levels of *Bmal1, Cry1, Dec1*, and *Rev-erb*α in the SCN were significantly decreased at some time points (Hayashi et al., 2013). The finding of normal PER2 expression in the SCN, but altered electrical output from the SCN, in a Parkinsonian (α-synuclein overexpressing) transgenic mouse further suggests disordered function or synchrony in the SCN (Kudo et al., 2011; Musiek, 2015). Therefore, the extent to which PD affects the SCN, and through which mechanisms, remains unclear.

MTPT-treated mice also had significantly lower locomotor activity during nocturnal hours and lower body temperatures (implying the involvement of the hypothalamus) during daytime and nighttime hours compared to the control mice (Hayashi et al., 2013). Peripheral hypothalamic areas, including the periventricular nucleus, demonstrate decreased expression of PER2 after the depletion of DA neurons with 6-hydroxydopamine (Gravotta et al., 2011). This suggests that DA depletion can affect circadian rhythm mechanisms differently in the SCN than in peripheral hypothalamic areas.

Hypothalamic DA neurons are also affected in PD, as is evidenced by both a 50% decrease in DA neurons in postmortem brains of PD patients (Conte-Devolx et al., 1985) and a decrease in DA levels of hypothalamic extracts from PD patients compared with control subjects (Pique et al., 1985). A PET study also showed a significant decrease in D_2_ receptor availability in the hypothalami of PD patients compared to that in control subjects (Politis et al., 2008). Hypothalamic DA degeneration may be related to the progression of PD, but earlier stages of the disease may not affect DA significantly. For example, plasma PRL levels of patients with early stages of idiopathic PD were not significantly different from the levels of the age-matched controls (Aziz et al., 2011), implicating proper function of DA from TIDA neurons. Conversely (and strange when considering that DA reduction should increase PRL levels), other patients with PD have shown significantly lower levels of plasma PRL (Murri et al., 1980; Winkler et al., 2002). Yet again, other PD patients have shown an increase in nocturnal PRL (Bellomo et al., 1991). These results provide evidence that hypothalamic DA neurons are affected with the progression of PD, with clear implications to normal circadian rhythms and potential disruption of PRL cycling. However, the exact mechanism as to how TIDA and other hypothalamic DA neurons are affected by the progression of the disease is not clear.

## Discussion

Circadian rhythms synchronize nearly every daily biological process – from gene transcription to behavior – and evidence of DA’s importance in the modulation of circadian rhythms is becoming more apparent. DA has been shown to have circadian-like activities in the retina, OB, striatum, midbrain, and hypothalamus, where it regulates, and is regulated by, clock genes in some of these areas. The various neuronal and molecular mechanisms reviewed here, and the mounting evidence that neurodegenerative diseases such as PD influence circadian rhythms, show that DA has both direct and indirect impacts on some of the circadian rhythms of the SCN and these peripheral brain areas (see Supplementary Table 1).

### Summary of Dopamine’s Involvement in Parkinson’s Disease and Associated Circadian Dysfunction

In addition to causing classic motor symptoms, reduced midbrain DA has been shown to disrupt circadian control of locomotor activity (Fifel and Cooper, 2014) and REM sleep (Lima et al., 2007) in PD. Reduced DA in the striatum due to nigrostriatal degeneration could contribute to RLS, blunt clock gene expression, and disrupt circadian rhythms in patients with PD (Turjanski et al., 1999; Videnovic and Golombek, 2013; Verwey et al., 2016).

Reduced DA levels in the retina observed in patients with PD (Nguyen-Legros, 1988; Harnois and Di Paolo, 1990; Adam et al., 2013) may contribute to visual symptoms (see Archibald et al., 2009) and impair light adaption (Prigge et al., 2016). As DA modulation of ipRGCs is involved in photic entrainment of the SCN (Gooley et al., 2001; Sakamoto et al., 2005), decreased retinal DA levels associated with PD may disrupt circadian rhythms.

Patients with PD often experience early olfactory dysfunction (Doty et al., 1988; Ross et al., 2008; Doty, 2012). Some olfactory dysfunction is linked to pathology and increased TH and DA levels in the OB (Del Tredici et al., 2002; Huisman et al., 2004; Mundinano et al., 2011), which may impair odor detection (Doty and Risser, 1989; Huisman et al., 2004) and odor discrimination (Tillerson et al., 2006) via actions at D_2_ receptors (Doty and Risser, 1989; Hsia et al., 1999; Berkowicz and Trombley, 2000; Ennis et al., 2001; Davila et al., 2003).

Mechanisms underlying circadian disruptions in PD remain unclear, but may involve afferent pathways to the SCN (e.g., impaired light transmission due to dopaminergic retinal degeneration) (Harnois and Di Paolo, 1990; Adam et al., 2013), the SCN itself (e.g., altered clock gene expression) (Hayashi et al., 2013; Mattam and Jagota, 2015), or downstream peripheral efferents (e.g., altered electrical output from the SCN) (Kudo et al., 2011; Videnovic and Golombek, 2013; Musiek, 2015). DA from other brain regions may exert feedback effects in the SCN via actions at D_1_ and D_5_ receptors (Rivkees and Lachowicz, 1997; Mendoza and Challet, 2014). Patients with PD have altered levels of PRL (Murri et al., 1980; Bellomo et al., 1991; Winkler et al., 2002), suggesting disruptions in PRL cycling involving TIDA neurons (Freeman et al., 2000; Bertram et al., 2010).

### New Directions in Research

New research should continue to explore how DA, as well as other neurotransmitters, affect(s) the circadian rhythms of the retina, OB, striatum, midbrain, and hypothalamus. Neurotransmitters serotonin and norepinephrine should also be studied, since the raphe nuclei and locus coeruleus neurons project throughout the brain (including the rat OB; Shipley et al., 1985; McLean and Shipley, 1987), and the activity of both transmitters fluctuates between sleep and wake cycles (Nolte, 2009; Corthell et al., 2013). Melatonin, a hormone that helps to induce sleep during darkness and regulates the release of DA in the retina (Doyle et al., 2002a), should continue to be studied in other brain areas to clarify how it interacts or regulates the other neurotransmitters. In addition to the brain areas discussed here, the circadian rhythms of other brain areas also may be influenced by DA’s activity. For example, the hippocampus receives a large dopaminergic input from the VTA via the mesolimbic pathway, and this input may regulate clock genes and proteins (McCarthy and Welsh, 2012), which could be involved in hippocampus-dependent learning (Ito and Schuman, 2007) and could protect against hippocampal cell death (Bozzi et al., 2000; Rocha et al., 2012; Bozzi and Borrelli, 2013).

## Conclusion

Circadian rhythms drive the daily biological processes, which in turn drive our daily lives. Disruptions in circadian rhythms – which can result from PD and other disorders as well as from environmental influences – can lead to various health risks and disorders (Schernhammer et al., 2006; Sookoian et al., 2007; Dochi et al., 2009; Dominguez-Rodriguez et al., 2009; McHill et al., 2014). As our understanding of how DA affects and is affected by circadian rhythms grows, new diagnostic methods (such as detection of hyposmia or anosmia as a precursor to circadian disruption) and pharmacologic tools may be developed to help those afflicted with disorders of circadian rhythms.

## Author Contributions

All authors contributed equally to this manuscript. KK wrote the first draft of the manuscript and the other authors, LB and PT, edited and contributed to all subsequent drafts. All authors agreed upon the final version of this manuscript.

## Conflict of Interest Statement

The authors declare that the research was conducted in the absence of any commercial or financial relationships that could be construed as a potential conflict of interest.

## Abbreviations

AC: adenylate cyclase
AC1: adenylate cyclase isoform 1
AHr: aryl hydrocarbon receptor
CRY: cryptochrome (clock gene and protein variant)
CT: circadian time
Cx36: Connexin 36 (gap junction channel)
D_1,2,3,4,5_: dopamine receptors 1, 2, 3, 4 and 5
DA: dopamine
DOPAC: 3,4-Dihydroxyphenylacetic acid
EPSC: excitatory postsynaptic current
ETC: external tufted cell (olfactory bulb)
GL: glomerular layer (olfactory bulb)
HVA: homovanillic acid
ipRGC: intrinsically photosensitive retinal ganglion cells
JGCs: juxtaglomerular cells (olfactory bulb)
M/TCs: mitral/tufted cells (olfactory bulb)
MPTP: 1-methyl-4-phenyl-1,2,3,6-tetrahydropyridine (neurotoxin that induces Parkinson’s-like symptoms)
NAcc: nucleus accumbens
NPAS2: neuronal PAS domain-containing protein 2 (clock gene and its protein variant)
OB: olfactory bulb
ONL: olfactory nerve layer (olfactory bulb)
OSN: olfactory sensory neuron (olfactory bulb)
P: postnatal day
PD: Parkinson’s disease
PER: period (clock gene and protein variant)
PET: positron emission tomography
PGC: periglomerular cell
PKA: protein kinase A
PRL: prolactin
RBD: REM sleep behavior disorder
REM: rapid-eye movement
RGC: retinal ganglion cells
RLS: restless leg syndrome
SAC: short axon cell (olfactory bulb)
SCN: suprachiasmatic nucleus
SN: substantia nigra (pars compacta and pars reticulata)
SNc: substantia nigra pars compacta
TH: tyrosine hydroxylase
TIDA: tuberoinfundibular dopamine neurons (hypothalamus)
VIP: vasoactive intestinal polypeptide
VTA: ventral tegmental area
ZT: Zeitgeber time.
