# Supplementary material for: Dopamine: A Modulator of Circadian Rhythms in the Central Nervous System
Source: Front Cell Neurosci. 2017 Apr 3;11:91. doi: 10.3389/fncel.2017.00091 (PMC5376559; doi:10.3389/fncel.2017.00091)
Supplement: Supplementary file 1 [file Table_1.docx]

Korshunov KS, Blakemore LJ, Trombley PQ*. “Dopamine: A Modulator of Circadian Rhythms in the Central Nervous System.”

Supplementary Material: **Table 1. Dopamine’s influence on the neuronal circadian rhythms of the five brain areas.**

| **Brain area** | **Retina** | **Olfactory Bulb** | **Striatum** | **Midbrain (includes VTA and SNc)** | **Hypothalamus (arcuate nucleus)** |
| --- | --- | --- | --- | --- | --- |
| DA neurons | Amacrine and/or interplexiform cells (species specific) (see Witkovsky 2004; Popova 2014). | PGCs or SACs (Baker 1986; Ennis et al. 200l; Kosaka and Kosaka 2009, 2016). | GABAergic striatal interneurons (Ibanez-Sandoval et al. 2010). | Mesolimbic, nigrostriatal, and mesocortical projecting neurons (see Russo and Nestler 2013; Nelson and Kreitzer 2014). | TIDA neurons and THDA neurons (see Freeman et al. 2000). |
| DA receptors | D_1_, D_2_, D_4_, and D_5_ receptors (see Witkovsky 2004; Beaulieu and Gainetdinov 2011; Popova 2014). | D_1_ (Nickell 1991; Liu et al. 2013) and D_2_ (Nickell et al. 1991; Hsia et al. 1999; Berkowicz and Trombley 2000; Ennis et al. 2001; Davila et al. 2003) receptors. | D_1_, D_2_, D_3_, and D_5_ receptors (see Beaulieu and Gainetdinov 2011). | D_1_ D_2_, and D_5_ receptors in the SN, D_2_ receptors in the VTA, D_3_ receptors in both areas (see Beaulieu and Gainetdinov 2011). | D_1_ and D_2_ receptors in arcuate nucleus (see Romero-Fernandez 2014). |
| Daily expression of TH and DA | DA content is high during daytime and low during nighttime (circadian and light dependent)(see Popova 2014). | Rodent DA content is high during the daytime and low during the nighttime (Corthell et al. 2013). | NAcc TH levels are rhythmically expressed and are highest during dark phase (Webb et al. 2009). DA, DOPAC, and HVA show daily rhythms of expression in the striatum (Castañeda et al. 2004). | SNc and VTA TH levels are rhythmically expressed and show peak expression at subjective day (Webb et al. 2009; Chung et al. 2014). MAOA, an enzyme that suppresses DA activity, and *Maoa* are rhythmically expressed in the VTA (Hampp et al. 2008). | DA turnover from TIDA neurons is highest in the morning, while PRL is highest during sleep (see Freeman et al. 2000). |
| Effects on neuronal circadian rhythms | D_4_ receptors may mediate circadian aspects of contrast sensitivity (Jackson et al. 2011, 2012; Hwang et al. 2013) and D_1_ receptors may mediate visual acuity (Jackson et al. 2012). D_4_ receptors and ipRGCs (needed for circadian photoentrainment) are involved in light adaption (see Witkovsky 2004; Prigge et al. 2016). | Diurnal variations in DA release (Corthell et al. 2013) may imply circadian activity of OB dopaminergic neurons (see Mendoza and Challet 2014), which may cause diurnal variation in olfactory sensitivity. | Rhythmic expression of NAcc TH likely contributes to the diurnal variation of sex reward (Webb et al. 2009). | Midbrain DA may be involved in maintenance of daily motor rhythms (Fifel and Cooper 2014). Also, rhythmic expression of VTA TH potentially contributes to the diurnal variation of drug (amphetamine) reward (Webb et al. 2009). | DA provides daytime inhibition of PRL release, resulting in cycling (see Freeman et al. 2000). |
| DA interactions with circadian (“clock”) genes and proteins | D_4_ receptors mediate the expression of the *Npas2* gene (Hwang et al. 2013). D_1_ receptors influence PER2 expression (Ruan et al. 2008). D_2_ receptors mediate the rhythmic expression of melanopsin mRNA by ipRGCs, (Sakamoto et al. 2005). | OB exhibits intrinsic circadian rhythms in clock gene (PER) activity in vitro (but unknown if linked to DA) (Granados-Fuentes et al. 2004). | Dorsal striatum’s PER2 expression depends on D_2_ receptor activation (Hood et al. 2010). D_1_, and/or D_2_, and D_3_ receptors regulate expression of *Per1*, *Clock*, *Bmal1*, and *NPAS2* in striatal cultured neurons (Imbessi et al. 2009). Rhythmic expression of PER1 and BMAL1 protein levels in the NAcc (Webb et al. 2009). | CLOCK (McClung et al. 2005; Sidor et al. 2015) and REV-ERBα (Chung et al. 2014) inhibit TH expression in the VTA. | PER1 and 2, expressed in TIDA neurons, are necessary for DA release (Sellix et al. 2006). |
| Relationship between DA and PD | IPL, where DA neurons are found, is decreased in PD (Hajee et al. 2009; Adam et al. 2013; Spund et al. 2013). Reduced retinal DA levels in PD may impair foveal vision (Bodis- Wollner 2009), contrast sensitivity (Bulens et al. 1989) and light adaption (see Archibald et al. 2009). | DA and TH neurons *increased* in the OBs of patients with PD (Huisman et al. 2004; Mundinano et al. 2011). This may contribute to hyposmia. Increased OB DA may impair odor detection (Doty and Risser 1989) and discrimination (Tillerson et al. 2006) in PD. | Reduced striatal DA transporters may contribute to RBD (Eisensehr et al. 2003) and disturbed striatal DA transmission may contribute to RLS (Turjanski et al. 1999), both common in PD. Nigrostriatal degeneration in PD may blunt clock gene expression and contribute to circadian disruptions (Videnovic and Golombek 2013; Verwey et al. 2016). | Degeneration of dopaminergic neurons in SN causes classic motor symptoms of PD (Carlsson 1972). Loss of midbrain DA impaired locomotor activity in MitoPark mouse model of PD (Fifel and Cooper 2014). Loss of SNc DA neurons impaired REM sleep in an MPTP rat model of PD (Lima et al. 2007). | Significant decrease in hypothalamic DA neurons in postmortem PD brains (Conte-Devolx et al. 1985) and a decrease in DA levels of hypothalamic extracts from PD patients (Pique et al. 1985). Patients with PD have altered levels of PRL (Murri et al. 1980; Bellomo et al. 1991; Winkler et al. 2002). |

*Corresponding author email: trombley@neuro.fsu.edu

**Abbreviations:** DA: dopamine; DOPAC: 3,4-Dihydroxyphenylacetic acid; GABA: γ-amino butyric acid; HVA: homovanillic acid; IPL: inner plexiform layer; ipRGCs: melanopsin-expressing intrinsically photosensitive retinal ganglion cells; MAOA: monoamine oxidase A; MPTP: 1-methyl-4-phenyl-1,2,3,6-tetrahydropyridine; NAcc: nucleus accumbens; OB: olfactory bulb; PD: Parkinson’s Disease; PGCs: periglomerular cells; PRL: prolactin; RBD: rapid-eye movement sleep behavior disorder; REM: rapid-eye movement; RLS: restless leg syndrome; SACs: short axon cells; SNc: substantia nigra pars compacta; TH: tyrosine hydroxylase; THDA neurons: tuberhypophyseal dopamine neurons; TIDA neurons: tuberoinfundibular dopamine neurons; VTA: ventral tegmental area.

**Table references**

**Adam, C.R., Shrier, E., Ding, Y., Glazman, S., and Bodis-Wollner, I.** (2013). Correlation of inner retinal thickness evaluated by spectral-domain optical coherence tomography and contrast sensitivity in Parkinson disease. *J Neuro-Ophthalmol*. 33, 137-142. doi: 10.1097/WNO.0b013e31828c4e1a.

**Archibald, N.K., Clarke, M.P., Mosimann, U.P., and Burn, D.J.** (2009). The retina in Parkinson's disease. *Brain*. 132(Pt 5), 1128-1145. doi: 10.1093/brain/awp068.

**Baker, H.** (1986). Species Differences in the Distribution of Substance P and Tyrosine Hydroxylase Immunoreactivity in the Olfactory Bulb. *J Comp Neurol*. 252, 206-226. doi: 10.1002/cne.902520206.

**Beaulieu, J.M., and Gainetdinov, R.R.** (2011). The physiology, signaling, and pharmacology of dopamine receptors. *Pharmacol Rev*. 63(1),182-217. doi: 10.1124/pr.110.002642.

**Bellomo, G., Santambrogio, L., Fiacconi, M., Scarponi, A.M., and Ciuffetti, G.** (1991). Plasma profiles of adrenocorticotropic hormone, cortisol, growth hormone and prolactin in patients with untreated Parkinson’s disease. *J Neurol*. 238(1), 19-22.

**Berkowicz, D.A. and Trombley, P.Q.** (2000). Dopaminergic modulation at the olfactory nerve synapse. *Brain Res*. 855(1), 90-99.

**Bodis-Wollner, I.** (2009). Retinopathy in Parkinson disease. *J Neural Transm*. 116: 1493-1501. doi: 10.1007/s00702-009-0292-z.

**Bulens, C., Meerwaldt, J.D., van der Wildt, G.J., and Keemink CJ.** (1989). Visual contrast sensitivity in drug-induced Parkinsonism. *J Neurol Neurosurg Psychiatry*. 52, 341–345.

**Carlsson, A.** (1972). Biochemical and pharmacological aspects of Parkinsonism. *Acta Neurol* *Scand Suppl*. 51, 11-42.

**Corthell, J.T., Stathopoulos, A.M., Watson, C.C., Bertram, R., and Trombley, P.Q.** (2013). Olfactory bulb monoamine concentrations vary with time of day. *Neuroscience*. 247, 234-241. doi: 10.1016/j.neuroscience.2013.05.040.

**Castañeda, T.R., de Prado, B.M., Prieto, D., and Mora, F.** (2004). Circadian rhythms of dopamine, glutamate and GABA in the striatum and nucleus accumbens of the awake rat: modulation by light. *J Pineal Res*. 36(3), 177-185.

**Chung, S., Lee, J.E., Yun, S., Choe, H.K., Park, S.B., Son, H.J., Kim, K.S., Dluzen, D.E., Lee, I., Hwang, O., Son, G.H., and Kim, K.** (2014). Impact of circadian nuclear receptor REV-ERBα on midbrain dopamine production and mood regulation. *Cell*. 157, 858-868. doi: 10.1016/j.cell.2014.03.039.

**Conte-Devolx, B., Grino, M., Nieoullon, A., Javoy-Agid, F., Castanas, E., Guillaume, V., Tonon, M.C., Vaudry, H., and Oliver, C.** (1985). Corticoliberin, somatocrinin and amine contents in normal and parkinsonian human hypothalamus. *Neurosci Lett*. 56(2), 217-222. doi: 10.1016/0304-3940(85)90132-6.

**Davila, N.G., Blakemore, L.J., and Trombley, P.Q.** (2003). Dopamine modulates synaptic transmission between rat olfactory bulb neurons in culture. *J Neurophysiol*. 90(1), 395-404. doi: 10.1152/jn.01058.2002.

**Doty, R.L. and Risser, J.M.** (1989). Influence of the D-2 dopamine receptor agonist quinpirole on the odor detection performance of rats before and after spiperone administration. *Psychopharmacology*. 98(3), 310-315. doi: 10.1007/BF00451680.

**Eisensehr, I., Linke, R., Tatsch, K., Kharraz, B., Gildehaus, J.F., Wetter, C.T., Trenkwalder, C., Schwarz, J., and Noachtar, S.** (2003). Increased muscle activity during rapid eye movement sleep correlates with decrease of striatal presynaptic dopamine transporters. IPT and IBZM SPECT imaging in subclinical and clinically manifest idiopathic REM sleep behavior disorder, Parkinson's disease, and controls. *Sleep*. 26(5), 507-512.

**Ennis, M., Zhou, F.M., Ciombor, K.J., Aroniadou-Anderjaska, V., Hayar, A., Borrelli, E., Zimmer, L.A., Margolis, F., and Shipley, M.T.** (2001). Dopamine D2 receptor-mediated presynaptic inhibition of olfactory nerve terminals. *J Neurophysiol*. 86(6), 2986-2997.

**Fifel, K. and Cooper, H.M.** (2014). Loss of dopamine disrupts circadian rhythms in a mouse model of Parkinson’s disease. *Neurobiol Dis*. 71, 359-369. doi: 10.1016/j.nbd.2014.08.024.

**Freeman, M.E., Kanyicska, B., Lerant, A., and Nagy, G.** (2000). Prolactin: Structure, function, and regulation of secretion. *Physiol Rev*. 80(4), 1524-1585.

**Granados-Fuentes, D., Saxena, M.T., Prolo, L.M., Aton, S.J., and Herzog, E.D.** (2004). Olfactory bulb neurons express functional, entrainable circadian rhythms. *Eur J Neurosci*. 19(4), 898-906. doi: 10.1111/j.0953-816X.2004.03117.x.

**Hajee, M.E., March, W.F., Lazzaro, D.R., Wolintz, A.H., Shrier, E.M., Glazman, S., and Bodis-Wollner, I.G.** (2009). Inner retinal layer thinning in Parkinson disease. *Ama Arch Opthalmol*. 127(6), 737-741. doi: 10.1001/archophthalmol.2009.106.

**Hampp, G., Ripperger, J.A., Houben, T., Schmutz, I., Blex, C., Perreau-Lenz, S., Brunk, I., Spanagel, R., Ahnert-Hilger, G., Meijer, J.H., and Albrecht, U.** (2008). Regulation of monoamine oxidase A by circadian-clock components implies clock influence on mood. *Curr Biol*. 18, 678-638. doi: 10.1016/j.cub.2008.04.012.

**Hood, S., Cassidy, P., Cossette, M.P., Weigl, Y., Verwey, M., Robinson, B., Stewart, J., and Amir, S.** (2010). Endogenous dopamine regulates the rhythm of expression of the clock protein PER2 in the rat dorsal striatum via daily activation of D_2_ dopamine receptors. *J Neurosci*. 30(42), 14046-14058. doi: 10.1523/JNEUROSCI.2128-10.2010.

**Hsia, A.Y., Vincent, J.D., and Lledo, P.M.** (1999). Dopamine depresses synaptic inputs into the olfactory bulb. *J Neurophysiol*. 82(2), 1082-1085.

**Huisman, E., Uylings, H.B.M., and Hoogland, P.V.** (2004). A 100% increase of dopaminergic cells in the olfactory bulb may explain hyposmia in Parkinson’s disease. *Movement Disord*. 19(6), 687-692. doi: 10.1002/mds.10713.

**Hwang, C.K., Chaurasia, S.S., Jackson, C.R., Chan, G.C.K., Storm, D.R., and Iuvone, P.M.** (2013). Circadian rhythm of contrast sensitivity is regulated by a dopamine-neuronal PAS-domain protein 2-adenylyl cyclase 1 signaling pathway in retinal ganglion cells. *J Neurosci*. 33(38), 14989-14997. doi: 10.1523/JNEUROSCI.2039-13.2013.

**Ibáñez-Sandoval, O., Tecuapetla, F., Unal, B., Shah, F., Koós, T., and Tepper, J.M.** (2010). Electrophysiological and morphological characteristics and synaptic connectivity of tyrosine hydroxylase-expressing neurons in adult mouse striatum. *J Neurosci*. 30(20), 6999-7016. doi: 10.1523/JNEUROSCI.5996-09.2010.

**Imbesi, M., Yildiz, S.,Arslan, A.D., Sharma, R., Manev, H., and Uz, T.** (2009). Dopamine receptor-mediated regulation of neuronal “clock” gene expression. *Neuroscience*. 158, 537-544. doi: 10.1016/j.neuroscience.2008.10.044.

**Jackson, C.R., Chaurasia, S.S., Hwang, C.K., and Iuvone, P.M.** (2011). Dopamine D_4_ receptor activation controls circadian timing of the adenylyl cyclase 1/cyclic AMP signaling system in mouse retina. *Eur J Neurosci*. 34, 57-64. doi: 10.1111/j.1460-9568.2011.07734.x.

**Jackson, C.R., Ruan, G.X., Aseem, F., Abey, J., Gamble, K., Stanwood, G., Palmiter, R.D., Iuvone, P.M., and McMahon, D.G.** (2012). Retinal dopamine mediates multiple dimensions of light-adapted vision. *J Neurosci*. 32(27), 9359-9368. doi: 10.1523/JNEUROSCI.0711-12.2012.

**Kosaka, T. and Kosaka, K.** (2009). Two types of tyrosine hydroxylase positive GABAergic juxtaglomerular neurons in the mouse main olfactory bulb are different in their time of origin. *Neurosci Res*. 64(4), 436-441. doi: 10.1016/j.neures.2009.04.018.

**Kosaka, T. and Kosaka, K.** (2016). Neuronal organization of the main olfactory bulb revisited. *Anat Sci Int*. (91), 115-127. doi: 10.1007/s12565-015-0309-7.

**Lima, M.M.S., Andersen, M.L., Reksidler, A.B., Vital, M.A.B.F., and Tufik, S.** (2007). The role of the substantia nigra pars compacta in regulating sleep patterns in rats. *PLoS ONE*. 2(6), e513. doi: 10.1371/journal.pone.0000513.

**Liu, S., Plachez, C., Shao, Z., Puche, A., and Shipley, T**. (2013). Olfactory bulb short axon cell release of GABA and dopamine produces a temporally biphasic inhibition-excitation response in external tufted cells. *J Neurosci*. 33(7), 2916-2926. doi: 10.1523/JNEUROSCI.3607-12.2013.

**McClung, C.A., Sidiropoulou, K., Vitaterna, M., Takahashi, J.S., White, F.J., Cooper, D.C., and Nestler, E.J.** (2005). Reulation of dopaminergic transmission and cocaine reward by the *Clock* gene. *Proc Natl Acad Sci U.S.A.* 102(26), 9377-9381. doi: 10.1073/pnas.0503584102.

**Mendoza, J., and Challet, E.** (2014). Circadian insights into dopamine mechanisms. *Neuroscience*. 282, 230-242. doi: 10.1016/j.neuroscience.2014.07.081.

**Mundinano, I.C., Caballero, M.C., Ordonez, C., Hernandez, M., DiCaudo, C., Marcilla, I., Erro, M.E., Tunon, M.T., and Luquin, M.R.** (2011). Increased dopaminergic cells and protein aggregates in the olfactory bulb of patients with neurodegenerative disorders. *Acta Neuropathol*. 122, 61-74. doi: 10.1007/s00401-011-0830-2.

**Murri, L., Iudice, A., Muratorio, A., Polleri, A., Barreca, T., and Murialdo, G.** (1980). Spontaneous nocturnal plasma prolactin and growth hormone secretion in patients with Parkinson’s disease and Huntington’s chorea. *Eur Neurol*. 19(3), 198-206. doi: 10.1159/000115147.

**Nelson, A.B. and Kreitzer, A.C.** (2014). Reassessing models of basal ganglia function and dysfunction. *Annu Rev Neurosci*. 37, 117-135. doi: 10.1146/annurev-neuro-071013-013916.

**Nickell, W.T., Norman, A.B., Wyatt, L.M., and Shipley, M.T.** (1991). Olfactory bulb DA receptors may be located on terminals of the olfactory nerve. *Neuroreport*. 2(1), 9-12.

**Pique, L., Jegou, S., Bertagna, X., Javoy-Agid, F., Seurin, D., Proeschel, M.F., Girard, F., Agid, Y., Vaudry, H., and Lutton, J.P.** (1985). Pro-opiomelanocortin peptides in the human hypothalamus: comparative study between normal subjects and Parkinson patients. *Neurosci Lett*. 54(2-3), 141-146.

**Popova, E.** (2014). Role of dopamine in distal retina. *J Comp Physiol A*. 200, 333-358. doi: 10.1007/s00359-014-0906-2.

**Prigge, C.L., Yeh, P.T., Liou, N.F., Lee, C.C., You, S.F., Liu, L.L., McNeill, D.S., Chew, K.S., Hattar, S., Chen, S.K., and Zhang, D.Q.** (2016). M1 ipRGCs influence visual function through retrograde signaling in the retina. *J Neurosci.* 36(27), 7184-7197. doi: 10.1523/JNEUROSCI.3500-15.2016.

**Romero-Fernandez, W., Borroto-Escuela, D.O., Vargas-Barroso, V., Narváez, M., Di Palma, M., Agnati, L.F., Larriva Sahd, J., and Fuxe, K.** (2014). Dopamine D1 and D2 receptor immunoreactivities in the arcuate-median eminence complex and their link to the tubero-infundibular dopamine neurons. *Eur J Histochem*. 58(3), 2400. doi: 10.4081/ejh.2014.2400.

**Ruan, G.X., Allen, G.C., Yamazaki, S., McMahon, D.G.** (2008). An autonomous circadian clock in the inner mouse retina regulated by dopamine and GABA. *PLOS Biol*. 6(10), 2248-2263. doi: 10.1371/journal.pbio.0060249.

**Russo, S.J. and Nestler, E.J.** (2013). The brain reward circuitry in mood disorders. *Nat Rev Neurosci*. 14, 609-625. doi: 10.1038/nrn3381.

**Sakamoto, K., Liu, C., Kasamatsu, M., Pozdeyev, N.V., Iuvone, P.M., and Tosini, G.** (2005) Dopamine regulates melanopsin mRNA expression in intrinsically photosensitive retinal ganglion cells. *Eur J Neurosci*. 22(12), 3129-3136. doi: 10.1111/j.1460-9568.2005.04512.x.

**Sellix, M.T., Egli, M., Poletini, M.O., McKee, D.T., Bosworth, M.D., Fitch, C.A., and Freeman, M.E.** (2006). Anatomical and functional characterization of clock gene expression in neuroendocrine dopaminergic neurons. *Am J Physiol Regul Integr Comp Physiol*. 290, R1309–R1323. doi: 10.1152/ajpregu.00555.2005.

**Sidor, M.M., Spencer, S.M., Dzirasa, K., Parekh, P.K., Tye, K.M., Warden, M.R., Arey, R.N., Enwright III, J.F., Jacobsen, J.P.R., Kumar, S., Remillard, E.M., Caron, M.G., Deisseroth, K., and McClung, C.A.** (2015). Daytime spikes in dopaminergic activity drive rapid mood-cycling in mice. *Mol Psychiatry*. 20, 1406-1419. doi: 10.1038/mp.2014.167.

**Spund, B., Ding, Y., Liu, T., Selesnick, I., Glazman, S., Shrier, E.M., and Bodis-Wollner, I.** (2013). Remodeling of the fovea in Parkinson disease. *J Neural Transm*. 120(5), 745-753. doi: 10.1007/s00702-012-0909-5.

**Tillerson, J.L., Caudle, W.M., Parent, J.M., Gong, C., Schallert, T., and Miller, G.W.** (2006). Olfactory discrimination deficits in mice lacking the dopamine transporter or the D2 dopamine receptor. *Behav Brain Res*. 172(1), 97-105. doi: 10.1016/j.bbr.2006.04.025.

**Turjanski, N., Lees, A.J., and Brooks, D.J.** (1999). Striatal dopaminergic function in restless legs syndrome: 18F-dopa and 11C-raclopride PET studies. *Neurology*. 52(5), 932-937.

**Verwey, M., Dhir, S., and Amir, S.** (2016). Circadian influences on dopamine circuits of the brain: regulation of striatal rhythms of clock gene expression and implications for psychopathology and disease. *F1000Res*. 24, 5. doi: 10.12688/f1000research.9180.1.

**Videnovic, A. and Golombek, D.** (2013) Circadian and sleep disorders in Parkinson's disease. *Exp Neurol*. 243, 45-56. doi: 10.1016/j.expneurol.2012.08.018.

**Webb, I.C., Baltazar, R.M., Wang, X., Pitchers, K.K., Coolen, L.M., and Lehman, M.N.** (2009). Diurnal variations in natural and drug reward, mesolimbic tyrosine hydroxylase, and clock gene expression in the male rat. *J Biol Rhythm*. 24(6), 456-476. doi: 10.1177/0748730409346657.

**Winkler, A.S., Landau, S., and Chaudhuri, K.R.** (2002). Serum prolactin levels in Parkinson’s disease and multiple system atrophy. *Clin Auton Res*. 12(5), 393-398. doi: 10.1007/s10286-002-0025-y.

**Witkovsky P.** (2004). Dopamine and retinal function. *Doc Ophthalmol*. 108, 17-40.
